# A checklist of vascular plants of Ewe-Adakplame Relic Forest in Benin, West Africa

**DOI:** 10.3897/phytokeys.175.61467

**Published:** 2021-04-12

**Authors:** Alfred Houngnon, Aristide C. Adomou, William D. Gosling, Peter A. Adeonipekun

**Affiliations:** 1 Association de Gestion Intégrée des Ressources (AGIR) BJ, Cotonou, Benin Association de Gestion Intégrée des Ressources Cotonou Benin; 2 Université d’Abomey-Calavi, Faculté des Sciences et Techniques Abomey-Calavi, Littoral, BJ, Abomey-Calavi, Benin Université d’Abomey-Calavi Abomey-Calavi Benin; 3 Institute for Biodiversity & Ecosystem Dynamics, University of Amsterdam, Amsterdam, the Netherlands University of Amsterdam Amsterdam Netherlands; 4 Laboratory of Palaeobotany and Palynology, Department of Botany, Lagos (Unilag), Nigeria Laboratory of Palaeobotany and Palynology, Department of Botany Lagos Nigeria

**Keywords:** Dahomey Gap, Flora, Kétou, Range-restricted species, Refugial

## Abstract

Covering 560.14 hectares in the south-east of Benin, the Ewe-Adakplame Relic Forest (EARF) is a micro-refugium that shows insular characteristics within the Dahomey Gap. It is probably one of the last remnants of tropical rain forest that would have survived the late Holocene dry period. Based on intensive field investigations through 25 plots (10 × 50 m size) and matching of herbarium specimens, a checklist of 185 species of vascular plant belonging to 54 families and 142 genera is presented for this forest. In addition to the name for each taxon, we described the life form following Raunkiaer’s definitions, chorology as well as threats to habitat. The Rubiaceae family was the richest (20 species) followed by the Fabaceae (15 species). Life forms showed the preponderance of phanerophytes (88%). The Chorological spectrum was dominated by Guineo-Congolean species (66%). Species richness estimated were 200.52 ± 9.2808 for *Bootstrap*; 217.62 ± 14.5972; 224.16 ± 15.3725 and 242.67 respectively for *Chao*, *Jacknife1* and *Jacknife2*. *Bootstrap* appears to be the estimation closer to the field records. In Benin, EARF is home for *Rinorea* species described as West African forest bio-indicators and single location for *Nesogordonia papaverifera*, *Mansonia altissima*, *Englerophytum oblanceolatum*, *Octolobus spectabilis*, *Vitex micrantha* and most of *Drypeteae* tribe species (*Drypetes aframensis*, *Drypetes afzelii*, *Drypetes gilgiana* and *Drypetes leonensis*) recorded in Benin. Our results provides baseline information for further in-depth analysis of vegetation history in Benin by raising the question on the past floristic connection of the Dahomey gap and community engagement in conservation.

## Introduction

At the continental level, African rain forests, primarily those of the Guineo-Congolean block, are the main centres of species diversity (White 1983; Lebrun 2001; Sosef et al. 2017; Droissart et al. 2018; Couvreur et al. 2021). However, in some regions of this block, landscape changes have been so severe that particular areas would have functioned as refugia, while diversity in surrounding areas would have experienced losses. This is probably the case with forest islands within the Dahomey Gap which is the dry corridor separating the West African rain forest into the Upper Guinean and Lower Guinean blocks (White 1983; Jenik 1994; Poorter et al. 2004). During the late Holocene dry period (3000–2500 yrs BP), the once continuous rain forest belt became fragmented and was reduced to isolated patches that would have persisted and survived as “small isolated humid pockets” (Dupont and Weinelt 1996; Salzmann and Hoelzmann 2005). In addition to these historical climatic oscillations, archaeologists and ecologists highlighted several proofs of human footprint such as metallurgy (cast iron), agriculture, pottery that would have gradually caused deforestation and may explain the phytogeographic status of the Dahomey gap in West Africa (Richards 1973; Paradis 1977; Wantchecon 1983; Brand 2001; Garnier et al. 2018). Indeed, this savanna intermingled with small forest patches within the Dahomey gap, is also seen as a cultural landscape, produced by humans for subsistence, security and/or worship uses of religious traditions (Juhé-Beaulaton 2010; Cousin 2018). Consequently, the vegetation of Benin which is supposed to be luxuriant is today largely dominated by farms, fallows and grasslands (Adjanohoun 1966; Jenik 1994; Sosef et al. 2017). One fifth of the original forests remain, fragmented into isolated patches (Poorter et al. 2004). Today, Benin is home to 2807 plant species in terms of floristic composition (Akoègninou et al. 2006). It is among the best explored botany countries of sampling completeness with 2460 species theoretically estimated between 2864 and 2889 species (Sosef et al. 2017). Despite this sampling effort, some species that have not yet been collected or reported may not be listed and therefore omitted. Either because they may have disappeared, following their habitat degradation, as was the case of *Chrysobalanus icaco* L. (Syn. *C. atacoriensis* A. Chev.), which would have disappeared following earthworks (Adjanohoun et al. 1989).

However in Benin, most of these remaining forest patches, although playing the role of a high conservation priority area for heritage plants, are still experiencing severe threats due to the lack of adequate conservation strategies (Oloukoï et al. 2007; Adomou et al. 2010). This is probably the case with Ewe and Adakplame Relic Forest (EARF) in the south east of Benin. Up to now, this relic still persists on a community land while showing insular characteristics with some rare and poorly known plant species. Earlier botanists who worked on this refugium include Chevalier (1910), Aubréville (1937) and Adjanohoun (1966). They noted the typical feature of the Ewe-Adakplame Relict Forest (EARF) described as a Timber-refuge of the time of tribal wars, as one having a considerable number of African rain forest species of Guineo-Congolean region comparable to those of Côte d’Ivoire (Adjanohoun et al. 1989, P. 20).

In this context, floristic details on EARF may be very useful for conservation purposes through restoration and rehabilitation of degraded land with native trees. Such information is necessary for further studies in biogeography and phylogeny on the one hand, to address the main speciation models and mechanisms that may apply across tropical Africa (Demenou et al. 2016, 2018; Couvreur et al. 2021), and on the other hand to reconstruct the history of Tropical Africa vegetation.

This paper aims to provide a comprehensive checklist of vascular plants occurring in EARF that will serve as baseline for understanding the history of this vegetation over millennia. By exploring the floristic composition of EARF, we can better appreciate the biogeographic status of some species previously reported by Achoundong (1996; 2000) as bio indicators in other West African forests located on either side of the Dahomey gap. The results could help (1) to better understand how the Dahomey Gap has affected the vegetation of this area and (2) to catalyze long-lasting conservation actions toward EARF.

## Methods area

### Study site

**Figure 1.**
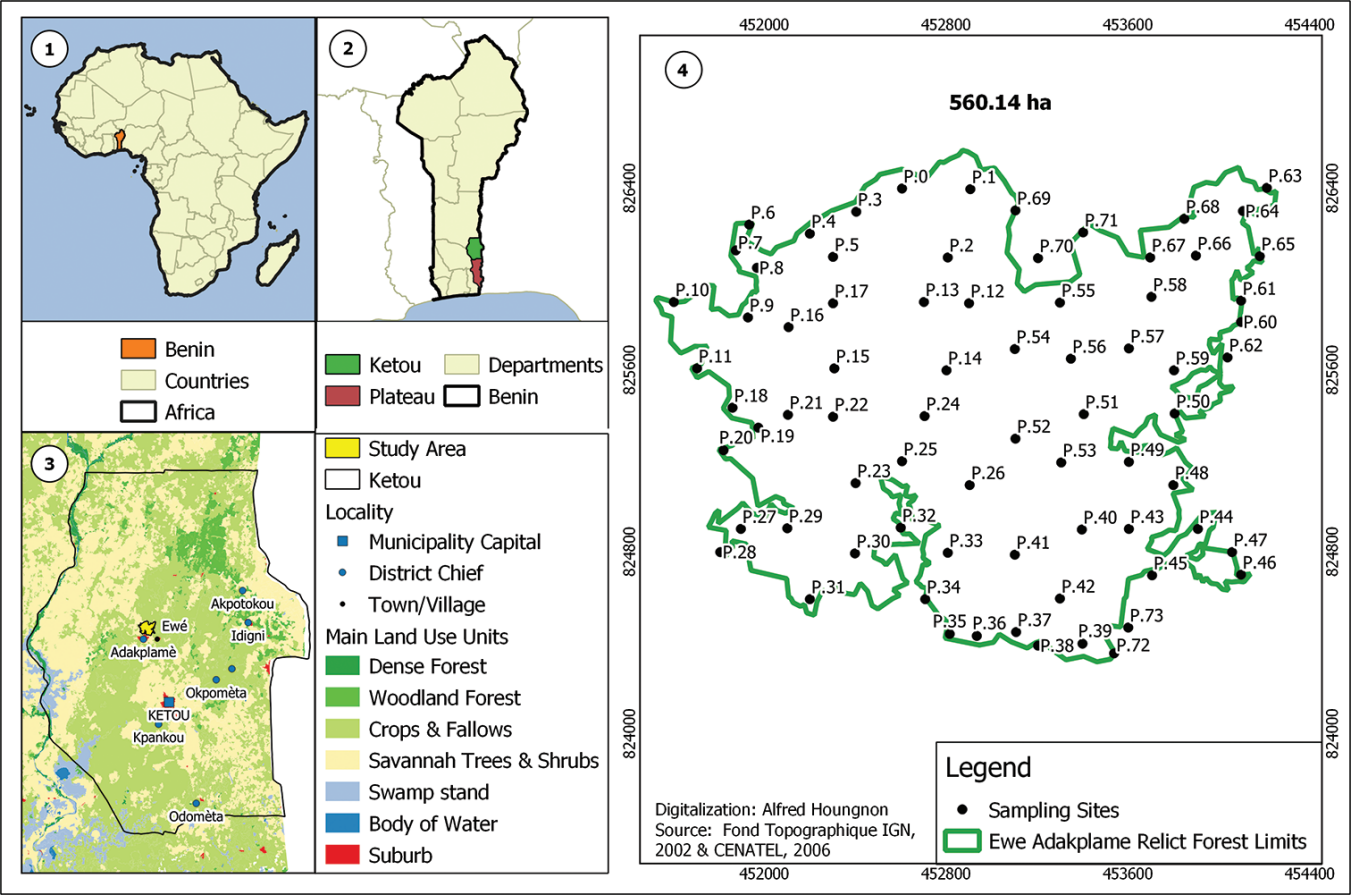
Location of the Ewe-Adakplame Relict Forest in Benin and positions of the sampling stands.

The EARF covers 560.14 hectares in the Kétou District in the south-east of Benin Republic at 07°27'59.195"N, 002°34'29.395"E (Fig. 1). This part of the country belongs to the Guineo-Congolean Region (White 1983; Adomou et al. 2006). The forest relic is located at the north-east of the depression of “Co” or “Lama” on the plateaus of low altitude that evolved on the pre-Cambrian base rocks (Adjanohoun et al. 1989). In Benin, the most important national protected areas are in the north. There are several other forests (albeit small) in the southern part of the country which are within the national protected areas network (e.g. gazetted forests of Dogo-Ketou, Pobe, Lama, Pahou) which are well-managed (Adomou et al. 2006). There is also the recent Transboundary biosphere reserve of Mono which is now part of the national protected areas network. However, EARF has not yet been included in this national protected areas network.

The mean annual rainfall in the EARF is between 900–1300 mm (Adjanohoun et al. 1989; CARDER 2002; Adomou et al. 2006) which contrasts to other similar African dense semi-deciduous forests. The rainfall recorded in Upper Guinea is between 1750–1900 mm (Martin 2008) in Côte d’Ivoire (West Africa) and annual rainfall measured around the Kakamega rain forest in East Africa was approximately 2215 mm (Cords 1987) and 1956 mm (Greiner 1991). Table 1 provides parameters such as temperature, relative humidity, vegetation and soil types of the study site. The landscape surrounding EARF is dominated by fallows, cultivation areas and housing. The vegetation is a mosaic of savanna with species of the Sudanian transition zone such as *Adansonia digitata* L., *Stereospermum kuntianum* Cham., *Trichilia emetic* Vahl, *Annona senegalensis* Pers., *Vitex doniana* Sweet, *Parkia biglobosa* (Jacq.) R.Br. ex G. Don, *Dichrostachys cinerea* (L.) Wight & Arn., *Pterocarpus erinaceus* Poir., *Pericopsis laxiflora* (Benth.) Meeuwen, *Daniellia oliveri* (Rolfe) Hutch. & Dalziel, *Vitellaria paradoxa* C.F. Gaertn., *Sarcocephalus latifolius* (Sm.) E.A. Bruce, *Uvaria chamae* P. Beauv., *Vitex grandifolia* Gürke and *Andropogon gayanus* Kunth (Biaou 2009; Armani et al. 2018). The total population of the villages of Ewe and Adakplame is 13,623 individuals with 2,078 households (INSAE 2016). The main activity is agriculture, followed by hunting, livestock breeding and local commerce.

**Table 1.** Ecological characteristics of the study region.

Location	6°25–7°30N	Adjanohoun et al. (1989)
		Adomou et al. (2006)
Annual rainfall	900–1100 mm	CARDER 2002, Adomou et al.(2006)
Rainfall trend	Bimodal	Adjanohoun et al. (1989)
Rainy season(s)	March–July & September–October	Adjanohoun et al. (1989)
		Adomou et al. (2006)
Dry season	August and November–February	Bani (2006)
Temperature	24–37 °C	CARDER (2002), Bani (2006)
Insolation	2135 h	CARDER (2002), Bani 2006
Relative humidity	78–95%	CARDER (2002), Bani (2006)
Climate type	Sub-equatorial	Adomou et al. (2006)
Length of plant growing season	240 days	CARDER (2002), Bani (2006)
Vegetation	Mosaic of Savanna	Adjanohoun et al. (1989)
		Adomou et al. (2006)
Soil types	Ferralitic soils without concretion	Adjanohoun et al. (1989), Bani (2006)
Altitude	200–286 m above sea level	Bani (2006)

### Sampling and data collection

The inventory of EARF plant species was conducted from February 2014 to December 2017. The forest investigation was based on a vegetation map divided into 250,000 m^2^ (500 × 500 m) grids following 6 transects, each of 500 m width and 3000 m length. Transects were oriented south-north. The floristic sampling covered different components of the EARF (Fig. 1). At each stand, a topometer (Chaining Buddy, Fremaco Devices, Canada) with disposable filament was used to delimit quadrats of 10 × 50 m. The observation stands were set out at intervals of 100 m along each transect line and there was one quadrat per plot of 250,000 m^2^. In total, 25 forest quadrats of 500 m^2^ were floristically surveyed. To set a preliminary list of EARF flora, species identification was first based on our self-background during the sampling field work with photo captures (Olympus Digital Camera SP-620 UZ Silver and Samsung Galaxy S7 Android 6.0.1). This approach was combined with description session (on field and at the National Herbarium). Voucher specimens were systematically collected for specimens whose determination is confused. They were compared with voucher specimens of the national Herbarium. To access the systematic information notes, the botanical nomenclature followed the Analytical Flora of Benin (Akoègninou et al. 2006). The list of plant species recorded was compared to online resources such as the “Catalog of life” (Hassler 2020) and the Benin National Red List (Neuenschwander et al. 2011) in order to access botanical information notes and the conservation status of species.

### Data analysis

The Angiosperm Phylogeny Group (APG IV 2009) and the legume subfamilies currently accepted by the legume phylogeny working group (LPWG 2017) were used to update the list of the vascular plants recorded in EARF. The taxonomic plant diversity was assessed in terms of species, genus, and family richness. The species richness (S) corresponds to the number of species recorded from sampling plots (*n* = 25). We used the functions “specpool” and “estimateR” in R software (R-Core-Team 2016) for the main reason that S is sensitive to sample size and this may introduce bias in our estimations based on the field record. To circumvent this, we use species accumulation curve and different estimation methods in order to appreciate in the case of our field study, the best estimator which is closest to our field record (Palmer 1990; Colwell and Coddington 1994; Chiu et al. 2014). This approach also helps to assess the completeness of our sampling effort. Chao, first order jackknife, second order jackknife and bootstrap were then used to estimate the total number of species surveyed and to draw species accumulation curves (R-Core-Team 2016; Oksanen et al. 2017).

Life forms assessment followed Raunkiaer (1934); Hutchinson and Dalziel (1954–1972): Ph: phanerophytes subdivided into meg: megaphanerophyte (> 30 m tall), mes: mesophanerophyte (8–30 m), mph: microphanerophyte (2–8 m), nph: nanophanerophyte (0.5–2 m); Ch: chamaephyte, Hc: hemicryptophyte; Th: therophyte; G: geophyte (Gb: with bulb, Gr: with rhizome and Gt: with tuber); Ep: epiphyte and their climbing forms L: liana (Lmph, Lnph and Lmes, LGr, LHc).

The Chorology types were established after Hutchinson and Dalziel (1954–1972) and White (1983), as follows: GC: Guineo-Congolean, SG: Sudano/Guinean transition, GE: Lower Guinean, GO: Upper Guinean, TA: Tropical Africa, AM: Afro-Malagasy, Pan: Pantropical.

### Data resources

The data underpinning the analysis reported in this paper are deposited in the Dryad Data Repository at https://doi.org/10.5061/dryad.z8w9ghxbg (Houngnon and Adomou 2021).

## Results

### Floristics

**Figure 2.**
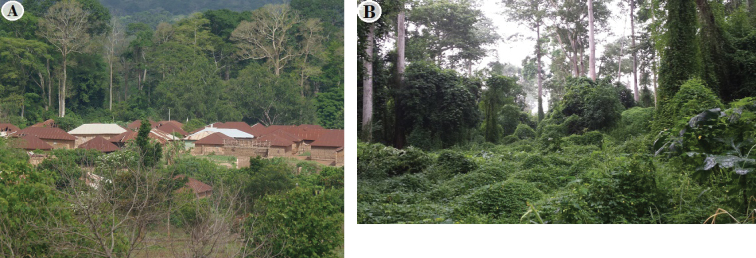
Panoramic view of Ewe-Adakplame Relict Forest **A** forest ecosystem in contact with Ewe village’s (Olympus photo A. Houngnon 2014) **B** forest gap with *Momordica charantia* carpet (Samung photo A. Houngnon 2016).

**Figure 3.**
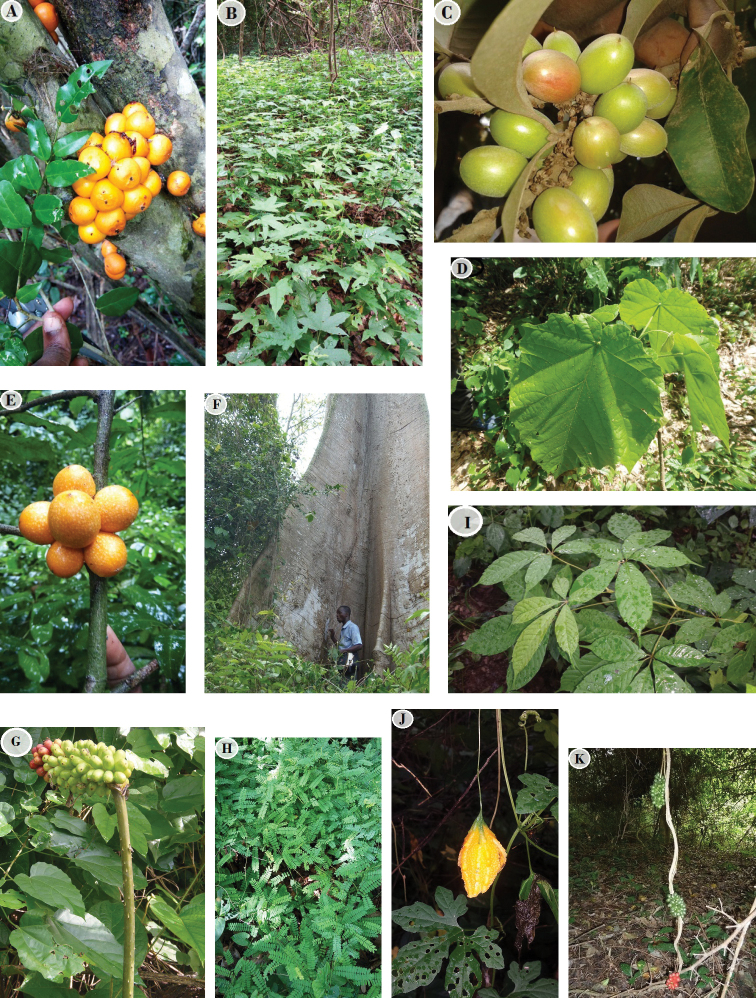
Common species of Ewe-Adakplame Relict Forest **A***Drypetes gilgiana* (Photo of Alfred Houngnon 2017) **B***Triplochiton scleroxylon* (Samsung photo A. Houngnon 2017) **C***Englerophytum oblanceolatum* (Olympus photo A. Houngnon 2014) **D***Mansonia altissima* (Olympus photo A. Houngnon 2014) **E***Uvariopsis tripetala***F***Ceiba pentandra* (Olympus photo A. Houngnon 2014) **G***Anchomanes welwitschii* (Samsung photo A. Houngnon 2015) **H***Abrus precatorius* (Samsung photo A. Houngnon 2016) **I***Vitex micrantha* (Samsung photo A. Houngnon 2016) **J***Momordica charantia* (Samsung photo A. Houngnon 2016) **K***Dioscoreophyllum cumminsii* (photo A. Adomou 2011).

Fig. 2A, B shows a panoramic view around and inside of EARF. Table 2 gives an overview of the vascular plant species recorded in the EARF. A total of 185 plant species distributed over 143 genera and 54 families was recorded (Table 2). With this figure, EARF conserves 6.59% of the national flora over 560.14 hectares of a community land. Of these, Rubiaceae was the most speciose family (20 species), followed by Fabaceae (15), Malvaceae (13), Apocynaceae (12), Sapindaceae (8) and Annonaceae (7). Capparaceae, Celastraceae, Dioscoreaceae, Putranjivaceae, Violaceae were each represented by five species. Among the 185 vascular plants of EARF 12% of the families (22) were represented by one species each. The genera *Dioscorea*, *Drypetes and Rinorea*, are represented by five species followed by genera *Albizia*, *Cissus*, *Strychnos* (4 species each) and *Celtis* and *Diospyros* (3 species each). EARF also provides habitat for *Chrysophyllum welwitschii*, (Not mentioned in the Flora), *Cissus glaucophylla* (Not mentioned in the Flora), *Drypetes aframensis*, (Not mentioned in the Flora), *Drypetes afzelii*, *Drypetes gilgiana*, *Drypetes leonensis*, *Englerophytum oblanceolatum*, *Mansonia altissima*, *Nesogordonia papaverifera*, *Octolobus spectabilis*, (Not mentioned in the Flora), *Rinorea batesii*, *Rinorea brachypetala* (Not mentioned in the Flora), *Rinorea dentata*, *Rinorea ilicifolia*, *Rinorea kibbiensis*, *Vitex micrantha*), (Not mentioned in the Flora) which appear to be confined to EARF that can be seen as the single location of their occurrence in Benin. Table 2 also describes the community data set using family, binomial, life-forms and Chorotypes. Some of these plant species are featured in Fig. 3A–K.

**Table 2.** Vascular plants of Ewe-Adapklame relict forest in Benin with their binomial, family life-forms and Chorotypes [Life-forms are meg: megaphanerophyte (> 30 m tall), mes: mesophanerophyte (8–30 m), mph: microphanerophyte (2–8 m), nph: nanophanerophyte (0.5–2 m); Ch: chamaephyte, Hc: hemicryptophyte; Th: therophyte; G: geophyte (Gb: with bulb, Gr: with rhizome and Gt: with tuber); Ep: epiphyte and their climbing forms L: liana (Lmph, Lnph and Lmes, LGr, LHc) and chorotypes are GC: Guineo-Congolean, SG: Sudano/Guinean transition, GE: Lower Guinean, GO: Upper Guinean, TA: Tropical Africa, AM: Afro-Malagasy and Pan: Pantropical].

Scientific name	Life forms	Chorology types	Voucher specimens
** Acanthaceae **			
Rhinacanthus virens (Nees) Milne. Readh. var. virens	Ch	GC	Houngnon 3860
** Amaranthaceae **			
*Cyathula prostrata* (L.) Blume	Th	Pan	Houngnon 3383
** Amaryllidaceae **			
Scadoxus multiflorus (Martyn) Raf. subsp multiflorus	Gb	TA	Houngnon 6724
** Anacardiaceae **			
Lannea nigritana (Sc. Elliot) Keay var. nigritana	mes	GO	De Souza 1971 a
*Spondias mombin* L.	mes	Pan	Maesen 7705
** Annonaceae **			
*Artabotrys dahomensis* Engl. & Diels.	Lnph	GE	Houngnon 97e
*Artabotrys velutinus* Sc. Elliot	Lnph	GC	Maesen 6612
*Monanthotaxis parvifolia* (Oliv.) Verdc.	Lnph	GE	Houngnon s.n.
*Monodora tenuifolia* Benth.	mph	GC	Éq. Bot. 105d
*Uvariodendron angustifolium* (Engl. & Diels) R.E.Fr	mph	GC	Houngnon 5571
*Uvariopsis tripetala* (Baker f.) G.E.Schatz Syn. *Dennettia tripetala* Baker f.	mph	GE	Akoègninou 2201
*Xylopia longipetala* De Wild. & T. Durand	mph	GC	Houngnon 4524
** Apocynaceae **			
*Alafia barteri* Oliv.	Lmph	GC	Chevalier 22841
*Ancylobotrys scandens* (Schumach. & Thonn.) Pifchon	Lmph	GC	Chevalier 23456
*Baissea zygodioides* (K. Schum.) Stapf	Lmph	GC	Houngnon 118c
*Cryptolepis nigrescens* (Wennberg) L. Joubert & Bruyns Syn. *Parquetina nigrescens* (Afzel.) Bullock	Lmph	GC	Le Testu 297
*Holarrhena floribunda* (G. Don) Dur. & Schinz	mph	TA	Houngnon 6574
*Hunteria umbellata* (K. Schum.) Hall. f. Syn. *H. eburnea* Pichon	mph	GC	Aké Assi 20284
*Landolphia hirsuta* (Hua) Pichon	Lmes	GC	Chevalier 23922
*Marsdenia latifolia* (Benth.) K. Schum.,	Lmph	TA	Akoègninou 5438
*Mondia whitei* (Hook. f.) Skeels	Lmph	TA	Adjakidjè 3007
*Motandra guineensis* (Thonn.) A. DC.	Lmph	TA	Adjanohoun 102
*Saba thompsonii* (A. Chev.) Pichon	Lmes	GC	Chevalier 22967
*Secamone afzelii* (Schultes) K. Schum.	Lmph	GC	Essou 3208
** Araceae **			
*Anchomanes difformis* (Blume) Engl. (Syn. *A. welwitschii* Rendle)	Gt	GC	Essou 1554
*Cercestis mirabilis* (N. E. Br.) Bogner Syn. *Rhektophyllum mirabile* N.E.Br.	Ep	GE	Akoègninou 3299.
** Aristolochiaceae **			
*Pararistolochia goldieana* (Hook. f.) Hutch. & Dalz.	LGr	GC	Houngnon 4605
** Asparagaceae **			
*Dracaena arborea* Bak	mph	GC	Maesen 6340
** Asteraceae **			
*Chromolaena odorata* (L.) R. King & H. Robinson	nph	AM	Sokpon B14
*Gymnanthemum coloratum* (Willd.) H. Rob. & B.Kahn	mph	SZ	Ayichédéhou 395
*Laggera crispata* (Vahl) Hepper & J. R. I. Wood.	Th	TA	Maesen 6746
** Bignoniaceae **			
*Newbouldia laevis* (P. Beauv.) Seem. ex Bureau	mph	GC	Houngnon 3087
** Boraginaceae **			
*Ehretia cymosa* Thonn.	mph	GC	Houngnon 5081
** Cannabaceae **			
*Celtis mildbraedii* Engl.	mes	GC	Essou 1648
*Celtis philippensis* Blanco Syn. *C. brownii* Rendle	mph	GC	Houngnon 2783
*Celtis zenkeri* Engl.	meg	GC	Sokpon 852
*Trema orientalis* Syn. *T. guineensis*	mph	GC	Houngnon 1714d
** Capparaceae **			
*Capparis brassii* DC. Syn. *C. thonningii* Schum.	Lnph	GC	Maesen 6701
Capparis erythrocarpos Isert var. erythrocarpos	nph	GC	Esson 1087
*Maerua duchesnei* (De Wild.) F. White Syn: *Ritcheia duchesnei* (De Wild.) Keay	mph	GC	Houngnon 229a
Ritchiea capparioides (Andr.) Britten var. capparoides	Lnph	GC	Houngnon 4200
*Ritchiea erecta* Hook. f. Syn. *R. pentaphylla* Gilg & Bened.	nph	GE	Aké Assi 20288
** Celastraceae **			
Loeseneriella africana (Willd.) N.Hallé var. africana Syn. Hippocratea Africana (Willd.) Loes.	Lmph	Pan	Houngnon 6573
*Reissantia indica* (Willd.) N. Hallé	Lnph	Pan	Akoègninou 4026
*Salacia longipes* (Oliv.) N. Hallé	nph	TA	Akoègninou 3291
*Salacia pallescens* Oliv.	nph	GC	Sokpon 2221
*Simicratea welwitschii* (Oliv.) Syn. *S. welwitschii* (Oliv.) N. Hallé	Lmph	GC	Essou 1467
** Combretaceae **			
*Combretum racemosum* P. Beauv.	Lmph	GC	Le Testu 191
** Commelinaceae **			
*Cyanotis lanata* Benth.	Ch	SG	Morton A4570
** Connaraceae **			
*Cnestis ferruginea* Vahl ex DC.	nph	GC	Houngnon 3051
*Cnestis corniculata* Lam. Syn. *Cnestis longiflora* Schellenb.	Lmph	GO	Chevalier 22828
*Rourea coccinea* (Bak.) Jongkind syn. *Byrsocarpus coccineus* Thonn. & Schumach.	nph	TA	Chevalier 22798b
** Convolvulaceae **			
*Calycobolus africanus* (G. Don) heine	Lnph	GC	Adjakidjè 4111
*Ipomoea mauritiana* Hall. f.	Lmph	Pan	Oumorou 740
** Cucurbitaceae **			
*Coccinia grandis* (L.) Voigt	Lnph	GC	De Souza & Paradis 444a
*Lagenaria breviflora* (Benth.) Roberty Syn. *Adenopus breviflorus* Benth.	Lmes	TA	Houngnon 443a, 1518a
*Luffa cylindrica* (L.) M. J. Roem syn. *Luffa aegyptiaca* Mill	Lnph	Pan	Houngnon 453a
*Momordica charantia* L.	Lnph	GC	Houngnon 1676
** Dichapetalaceae **			
*Dichapetalum madagascariense* Poir. Syn. *D guineense* (DC.) Keay	Lmph	GC	Adomou 95
*Tapura fischeri* Engl.	mph	GC	Houngnon 1878a
** Dioscoreaceae **			
*Dioscorea bulbifera* L. var. bulbifera	Gt	Pan	Essoun 3316
*Dioscorea lecardii* De Wild.	Gt	SZ	Pauwels 8139
*Dioscorea odoratissima* Pax Syn. *D. praehensilissensu* F.T.A, F.W.T.A	Gt	SG	Chevalier 24154
*Dioscorea quartiniana* A. Rich.	Gt	SZ	Sokpon 2329
*Dioscorea sagittifolia* Pax syn. *D. abyssinica* Hochst. ex Kunth	Gt	SZ	Paradis et Houngnon: 619d
** Ebenaceae **			
*Diospyros abyssinica* (Hiern) White	mes	GC	Houngnon 627b
*Diospyros monbuttensis* Gürke	mph	GC	Houngnon 629c
*Diospyros soubreana* F. White	nph	GC	Houngnon 2824
** Euphorbiaceae **			
*Erythrococca anomala* (Juss. ex Poir.) Prain	nph	GC	Houngnon 3345
Mallotus oppositifolius (Geisel.) Müell. Arg. var. oppositifolius	nph	AM	Adjakidjè & Akoègninou 590c
*Tragia senegalensis* Müll. Arg.	Lnph	SG	Adjakidjè 2803
** Fabaceae **			
**Caesalpinoideae (Mimosoid clade)**			
*Acacia pennata* (L.) Willd.	Lmph	TA	Essou 1672
Acacia polyacantha Willd. subsp. Campylacantha (Hochst. ex A. Rich.) Brenan	mes	SZ	Maesen 6703
Albizia adianthifolia (Schum.) W. Wight var. adianthifolia	mes	GC	Adjakidjè 4163
*Albizia glaberrima* (Schum. & Thonn.) Benth.	mph	GC	Houngnon 6532
*Albizia ferruginea* (Guill. & Perr.) Benth.	mes	GC	Paradis & Houngnon 933a
*Albizia zygia* (DC.) J. F. Macbr.	mes	GC	Houngnon 936d
*Mezoneuron benthamianum* (Baill.) Herend. & Zarucchi	Lmph	GC	Paradis & Houngnon 277c
** Detarioideae **			
*Detarium senegalense* J.F. Gmel.	mes	GC	Houngnon 268^e^
**Dialioideae**			
*Dialium guineense* Willd.	mes	GC	Spire 118
**Faboideae / Papilionoideae**			
*Abrus precatorius* L.	Lnph	Pan	Houngnon 1423g
*Dalbergia lactea* Vatke	Lmph	GE	De Souza & Paradis 1239e
*Dalbergia melanoxylon* Guill. Perr.	mph	SG	Adomou 167
Desmodium salicifolium (Poir.) DC. var. salicifolium	nph	GC	Frahm-Leliveld 57139
*Dolichos trilobus*	Lnph	SZ	Adomou 80
*Millettia thonningii* (Schum. & Thonn.) Bak.	mph	GC	Essou 1164
** Icacinaceae **			
*Stachyanthus occidentalis* (Keay & Miège) Boutique syn. *Neostachyanthus occidentalis* Keay & Miège	Lnph	GO	Essou 1102
** Lamiaceae **			
*Clerodendrum capitatum* (Willd.) Schum. & Thonn.	Lmph	GC	Lisowski 0-929
*Hoslundia opposita* Vahl	nph	AM	Pauwels 8286
*Premna quadrifolia* Schum. & Thonn.	nph	GO	Sokpon 1068
*Vitex micrantha* Gürke*	mes	GC	Adomou s.n.
** Linaceae **			
*Hugonia platysepala* Welw. ex Oliv.	Lmph	GC	Paradis & Houngnon 831a
** Loganiaceae **			
*Strychnos barteri* Soler.	Lmes	GC	Paradis & Houngnon 838a
*Strychnos floribunda* Gilg	Lmes	GC	Maesen 6821
*Strychnos nigritana* Bak.	Lmes	GC	Akoègninou 3289
*Strychnos splendens* Gilg	Lmes	GC	Houngnon 835b
** Malvaceae **			
*Abutilon mauritianum* (Jacq.) Medic.	Ch	TA	De Souza & Paradis 851a
*Ceiba pentandra* (L.) Gaertn.	meg	Pan	Houngnon 188a
*Hibiscus lunariifolius* Willd.	Lmph	Pan	Adomou s.n.
*Hibiscus owariensis* P. Beauv.	nph	GC	Paradis et Houngnon 856a
*Cola milfenii* K. Schum.	mph	GC	Houngnon 4399
*Glyphaea brevis* (Spreng.) Monachino	mph	GC	Houngnon 2036^e^
*Grewia carpinifolia* Juss.	mph	GC	Houngnon 1446f
Mansonia altissima (A. Chev.) A. Chev. var. altissima*	mes	GC	Houngnon 1309a ; 4322
*Nesogordonia papaverifera* (A. Chev.) syn *N. kabengaensis* (K.Schum.)*	mph	GC	Houngnon 13l0a
*Octolobus spectabilis* Welw. Syn. *O. angustatus* Hutch.*	nph	GC	Adomou s.n.
*Pterygota macrocarpa* K. Schum *.	mph	GC	Houngnon 4321
*Sterculia tragacantha* Lindl.	mes	GC	De Souza & Houngnon 188d
*Triplochiton scleroxylon* K. Schum.	meg	GC	Chevalier 22819
** Melastomataceae **			
Memecylon afzelii G. Don var. afzelii	Lnph	GC	Houngnon 897c
*Warneckea memecyloides* (Benth.) Jac. Fél Syn. *Memecylon memecyloides* (Benth)	Lmph	GC	De Souza & Paradis 900a
** Meliaceae **			
Trichilia prieureana A. Juss. subsp. prieureana	mph	GC	Adomou 90
** Menispermaceae **			
*Dioscoreophyllum cumminsii* (Stapf) Diels	Lnph	GC	Houngnon 919a
*Rhigiocarya racemifera* Miers	Lnph	GC	Maesen 6820
*Tiliacora funifera* (Miers) Oliv.	Lmph	GC	De Souza 92li
*Triclisia subcordata* Oliv.	Lnph	GC	Sokpon 31
** Moraceae **			
*Antiaris toxicaria* Lesch.	meg	GC	Essou 1547
*Ficus recurvata* De Wild. Syn. *Ficus goliath* A. Chev.	mes	GC	Adomou s.n.
*Ficus ovata* Vahl,	Ep	GC	Adomou s.n.
*Milicia exelsa* (Welw.) Berg Syn. *Chlorophora excelsa* (Welw.) benth.	meg	GC	Chevalier 23169
** Olacaceae **			
Olax subscorpioidea Oliv. var. subscorpioidea	mph	GC	Houngnon 7652
** Oleaceae **			
*Schrebera arborea* A. Chev.	mes	GC	Akoègninou 2187
** Opiliaceae **			
*Opilia amentacea* Roxb. Syn. *O. celtidifolia* (Guill. & Perr) Endl.,	Lmph	SZ	Adjakidjè 1477
** Pandaceae **			
*Microdesmis keayana* J. Léonard, syn. *M. puberula* Hook. f.	mph	GC	Pauwels 8298
** Passifloraceae **			
*Adenia cynanchifolia* (Benth.) Harms	Lmph	GE	Adomou s.n.
*Adenia lobata* (Jacq.) Engl.	Lmph	GC	Essou 1637
** Phytolaccaceae **			
*Hilleria latifolia* (Lam.) H. Walt.	Ch	AM	Adomou 129
** Poaceae **			
*Acroceras gabunense* (Hack.) Clayton*	Th	GC	Mission *ACCT/Bénin 2165*
*Olyra latifolia* L.	nph	GC	Houngnon 720c
Oplismenus hirtellus (L.) P. Beauv. subsp. Hirtellus	Ch	SG	De Souza & Paradis 722a
*Streptogyna crinita* P. Beauv.	Gr	GC	Houngnon 765b
** Polygalaceae **			
*Carpolobia lutea* G. Don	mph	GC	Maesen 6617
** Putranjivaceae **			
*Drypetes aframensis* Hutch.*	mph	GO	Adomou s.n.
*Drypetes afzelii* (Pax) Hutch.,*	mes	GO	Houngnon 177 la
*Drypetes floribunda* (Müll. Arg.) Hutch.	mph	GC	Houngnon 4266
*Drypetes gilgiana* (Pax) Pax & Hoffm.*	nph	GC	Akoègninou 2196
*Drypetes leonensis* Pax,*	mes	GC	Houngnon 1771b
** Rhamnaceae **			
*Lasiodiscus mannii* Hook. f.	mph	GC	Houngnon 1329b
** Rubiaceae **			
*Aidia genipiflora* (DC.) Dandy	mph	GC	Maesen 6611
*Chassalia kolly* (Schumach.) Hepper	nph	GC	Maesen 6358
*Coffea ebracteolata* (Hiern) Brenan	Lnph	GC	Lejoly&Ganglo 2
*Cremaspora triflora* (Thonn.) K. Schum.	Lmph	GC	Maesen 6284
*Leptactina involucrata* Hook. f.	Lnph	GC	Adomou s.n.
*Gardenia nitida* Hook.	mph	GC	Adomou 73
*Hymenodictyon floribundum* (Steud. & Hochst.) B.L.Rob.	mes	GC	Sinsin 2863
*Keetia hispida* (Benth.) Bridson	Lmph	GC	Adomou s.n.
*Morinda lucida* Benth.	mph	Pan	Maesen 6651
*Oxyanthus pallidus* Hiern	nph	GC	Adomou s.n.
Oxyanthus speciosus DC. subsp. speciosus	nph	GC	Essou 2496
*Pavetta corymbosa* (DC.) F. N. Williams	mph	SG	Sokpon 1884
*Pouchetia africana* DC.	nph	GC	Houngnon 6659
*Psydrax horizontalis* (K. Schum. & Thonn.) Bridson	Lmph	SG	Maesen 6710
*Psydrax parviflora* (Afzel.) Bridson	nph	GO	Maesen 6287
*Rothmannia longijlora* Salisb	mph	GC	Le Teslu 101
*Rothmannia urcelliformis* (Hiern) Bullock ex Robyns	mph	GC	Dansi TW 50799
*Rytigynia canthioides* (Benth.) Robyns	mph	GC	Adomou s.n.
*Vangueriella nigerica* (Robyns) Verdc. Syn. *Vangueriopsis nigerica* Robyns	mph	SZ	Maesen 6315
*Vangueriella spinosa* (Schumach.&Thonn.)Verdc. Syn. *Vangueriopsis spinosa* Hepper	mph	SZ	Adomou 32
** Rutaceae **			
*Zanthoxylum leprieurii* Guill. & Perr. Syn. *Fagara angolensis* Engl.	mph	GC	Houngnon 535a
*Zanthoxylum zanthoxyloides* (Lam.) Zepernick & Timber	mph	SG	Essou 2396
** Salicaceae **			
*Dovyalis zenkeri* Gilg (+) Syn. *D. afzelii* Gilg. (+)	nph	GO	Houngnon 1364a
*Flacourtia indica* (Burm. f.) Merr. Syn. *Flacourtia flavescens* Willd.	mph	GC	Houngnon 6606
** Sapindaceae **			
*Allophylus africanus* P. Beauv.	mph	GC	Houngnon 4037
*Allophylus spicatus* (Poir.) Radlk.	mph	GC	Houngnon 4037
*Blighia sapida* Koenig	mPh	Pan	Houngnon 5472
*Blighia unijugata* Bak.	mph	GC	Paradis & Houngnon 1693d
*Deinbollia pinnata* (Poir.) Schumach. & Thonn.	nph	GC	Maesen 6397
*Lecaniodiscus cupanioides* Planch.	mph	GC	Maesen 6310
*Majidea forsteri* (Sprague) Radlk.	meg	GC	Houngnon 1254a
*Pancovia bijuga* Willd.	mph	GC	Houngnon 4978
** Sapotaceae **			
*Chrysophyllum welwitschii* Engl.* (+)	Lnph	GC	Adomou s.n.
*Englerophytum oblanceolatum* (S.Moore) T.D.Penn. syn. Bequaertiodendron oblanceolatum* (S.Moore) Heine & J. H. Hemsl.	nph	TA	Maesen 6154
*Pouteria alnifolia* (Baker) Roberty Syn. *Malacantha alnifolia* (Baker)	mph	GC	Sokpon 1915
** Smilacaceae **			
*Smilax anceps* Willd.Syn. *S. kraussiana* Meissner	LGr	TA	Chevalier 24225
** Solanaceae **			
*Solanum terminale* Forssk. Subsp inconstans (C.H. Wright) Heine	Lmph	GC	Yédomonhan 173
** Ulmaceae **			
*Chaetachme aristata* Planch.	mph	GC	Houngnon 1784c
** Violaceae **			
*Rinorea batesii* Chipp, Kew Bull. 293 (1923).	nph	GC	Adomou 83
*Rinorea brachypetala* (Turcz.) Kuntze	nph	GC	Adomou s.n.
*Rinorea dentata* (P.Beauv.) Kuntze	mph	GC	Houngnon
*Rinorea ilicifolia* (Welw. ex Oliv.) Kuntze *	nph	GC	Adomou 109
*Rinorea kibbiensis* Chipp.	nph	GC	Paradis & Houngnon 1347a
** Vitaceae **			
*Cissus glaucophylla* Hook. f.	Lnph	GC	Adomou s.n.
*Cissus petiolata* Hook. f.	Lnph	GC	Adjakidjè 2976
Cissus populnea Guill. & Perr. var. populnea	LHc	SZ	Houngnon 68d
*Cissus quadrangularis* L.	Lmph	SZ	Houngnon 5105

*: Plant species restricted to Ewe Adakplame Relict Forest (+): Non recorded

### Life form spectrum

**Figure 4.**
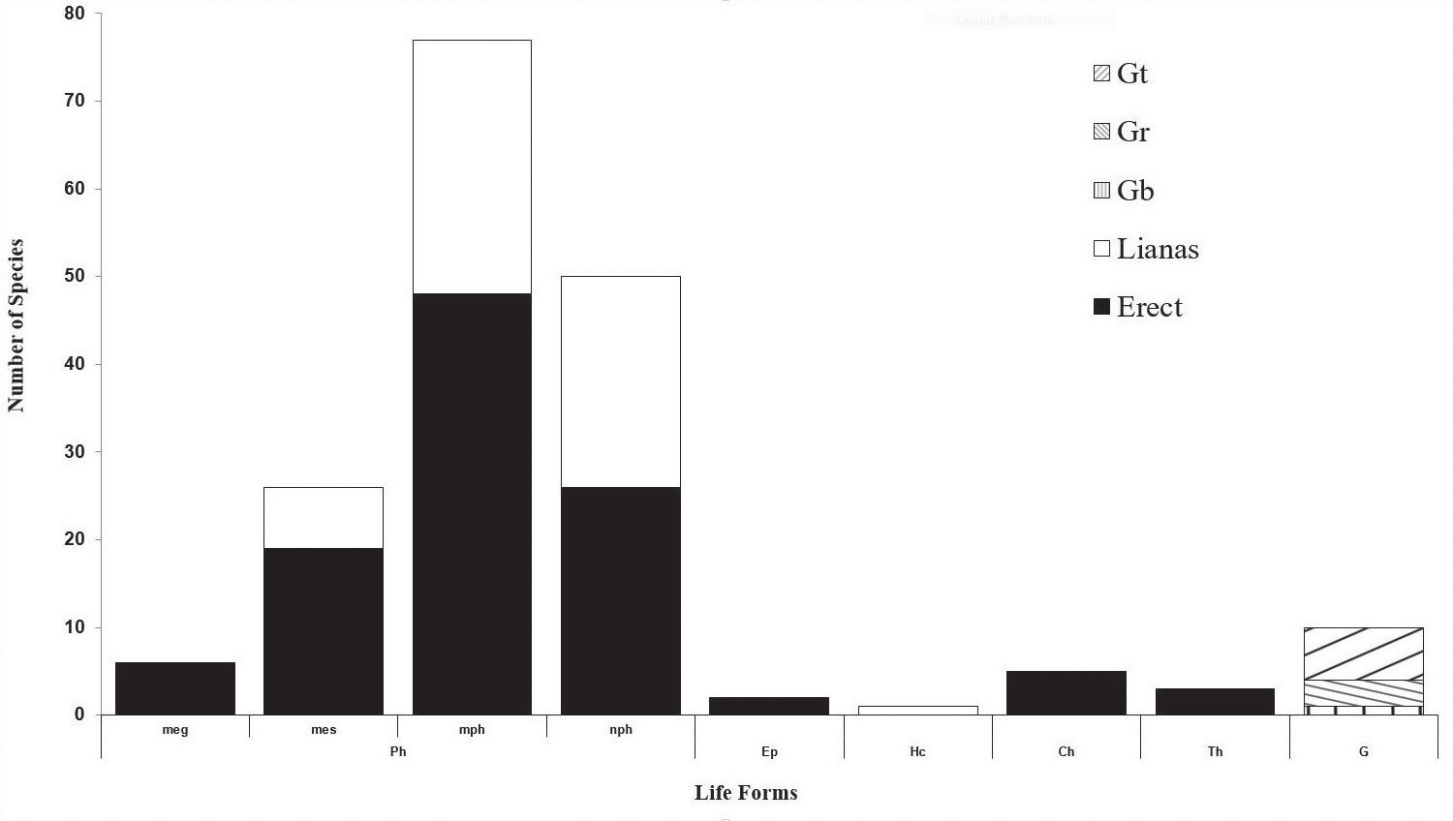
Life form spectrum of the Ewe-Adakplame relict forest. Erects are represented by Ph: Phanerophytes including megaphanerophytes (meg), mesophanerophytes (mes), microphanerophytes (mph), nanophanerophytes (nph), G: Geophytes are: Gb: with bulb, Gr: with rhizome and Gt: with tuber Ch: Chamaephytes, Th: Therophytes, Ep: Epiphytes, Hc: Hemicryptophytes. Climbing forms are L: Lianas (mph, nph and mes, Gr, Hc).

The most common life forms were phanerophytes (88%), containing, 3% of mega phanerophytes (meg) which are very large forest trees, 14% of mesophanerophytes (mes) or medium-sized forest trees, 43% of microphanerophytes (mph) or small forest trees and 28% of shrubs gathered into nanophanerophytes (nph). We recorded 33% of lianas, 6% of geophytes and 6% for chamaephyte, therophyte, epiphyte, hemicryptophyte (Fig. 4). The microphanerophytes were most representative among phanerophytes. The tree layer was discontinuous and composed of *Celtis mildbraedii* (Cannabaceae), *Triplochiton scleroxylon* (Malvaceae), *Antiaris toxicaria* (Moraceae), *Celtis zenkeri* (Cannabaceae), *Dialium guineense* (Fabaceae), *Ceiba pentandra* (Malvaceae), *Mansonia altissima* (Malvaceae), *Milicia exelsa* (Moraceae), and *Nesogordonia papaverifera* (Malvaceae).

### Chorology of Ewe-Adakplame relict forest

**Figure 5.**
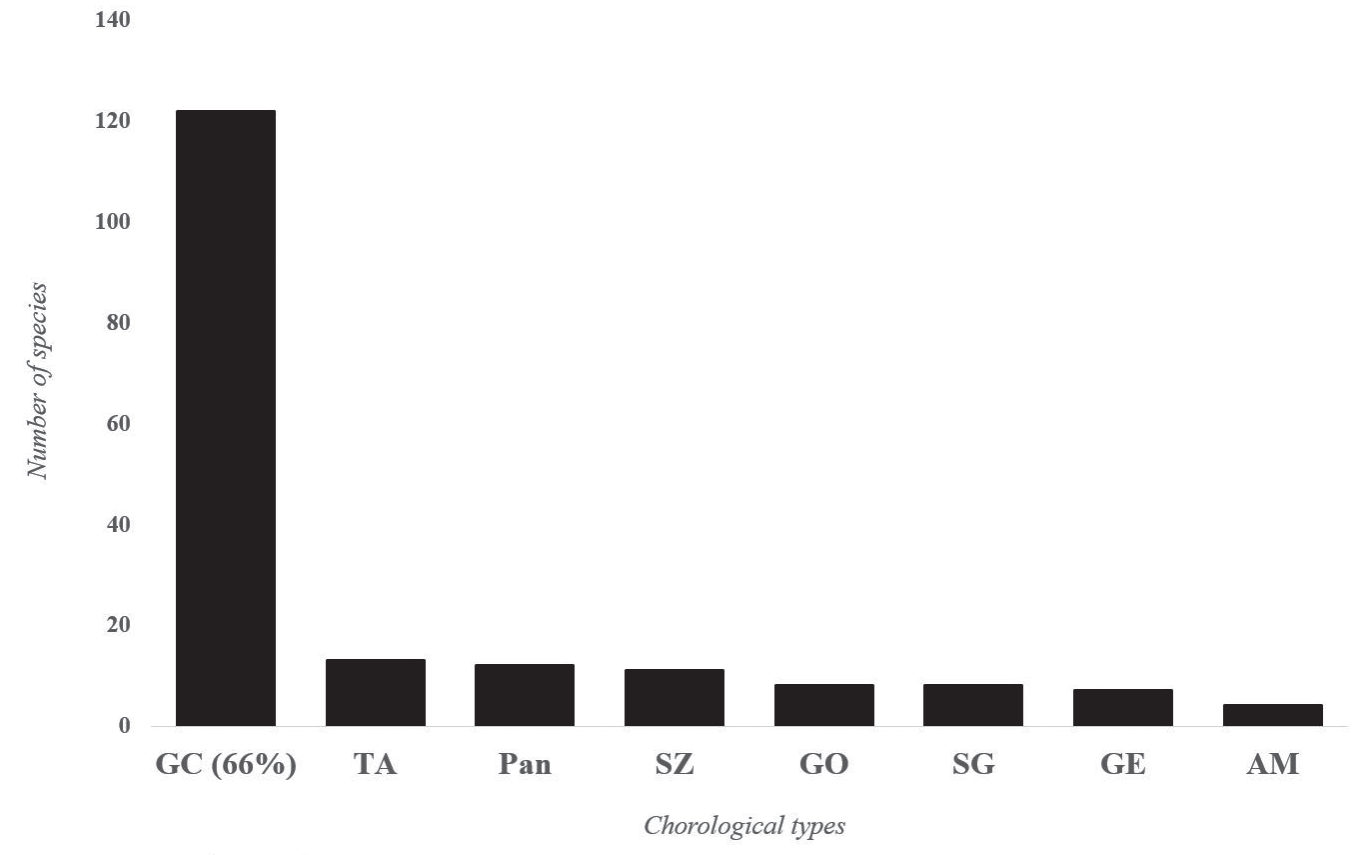
Chorological spectrum of the Ewe-Adakplame Relict Forest. GC: Guineo-Congolean, SG: Sudano/Guinean transition, GE: Lower Guinean, GO: Upper Guinean, TA: Tropical Africa, AM: Afro-Malagasy and Pan: Pantropical.

The most representative chorotypes (Fig. 5) included Guineo-Congolean species (66%), followed by 14% of wide distribution species including Tropical Africa (TA) and Pantropical (Pan) plant species. Upper Guinea species included plants such as *Uvariopsis tripetala* Syn. *Dennettia tripetala* (Annonaceae), *Drypetes aframensis* (Putranjivaceae tribe Drypeteae), *Stachyanthus occidentalis* Syn. *Neostachyanthus occidentalis* (Icacinaceae), Lannea nigritana var. nigritana (Anacardiaceae), *Psydrax parviflora* (Rubiaceae), *Premna quadrifolia* (Lamiaceae), *Cnestis corniculata* (Connaraceae). *Monanthotaxis parvifolia* (Annonaceae), *Artabotrys dahomensis* (Annonaceae), *Dalbergia lactea* (Fabaceae – Faboideae), *Ritchiea erecta* Syn. *R. pentaphylla* (Capparaceae) and *Cercestis mirabilis* Syn. *Rhektophyllum mirabile* (Araceae) are among Lower Guinea species.

### Species richness estimations

**Figure 6.**
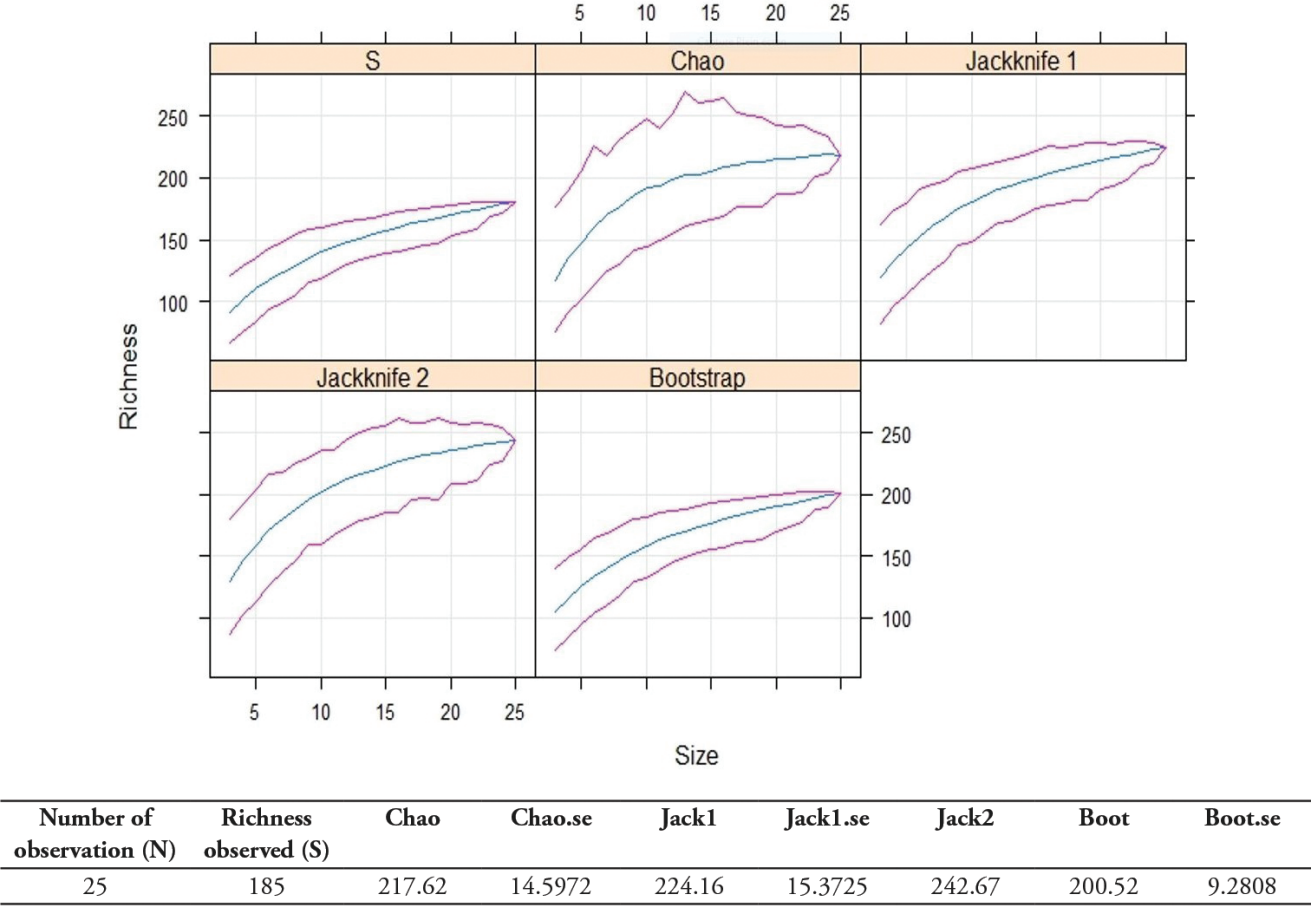
Species richness and richness estimations (Chao, first order jackknife, second order jackknife and bootstrap) (y-axis) in relation to sample size (x-axis) at the Ewe-Adakplame relict forest.

The counted number of plant species for the EARF was 185. This corresponds to the species richness (S) or the number of species that has been recorded from plot sampling and listed in Table 2. The species richness estimations as per *Bootstrap*, *Chao*, *Jacknife1* and *Jacknife2* were respectively 200.52 ± 9.2808; 217.62 ± 14.5972, 224.16 ± 15.3725 and 242.67. We can see that the species richness estimates differ strongly giving a range of 200.52–242.67 species. The species accumulation curves in Fig. 6 show that they were hardly tending towards the asymptote and are still climbing at the right-hand end signifying that the sampling effort was insufficient. This suggests that the sampling has not captured nearly all the species in EARF and that many species were missed (on average approximately 16 species (± 9.2808) for *Bootstrap*, 33 species (± 14.5972) for *Chao*, 39 species (± 15.3725) for *Jacknife1* and 58 species for *Jacknife2*.

## Discussion

The Ewe-Adakplame Relict Forest corresponds to the semi-deciduous forest type, which was described in Benin as the only *Drypetes aframensis*-*Nesogordonia papaverifera* community (Adomou et al. 2009). Guineo-Congolean species are abundant (66%) although the EARF is located in a matrix of savanna-dominated vegetation. Guineo-Congolean species accounted for 33% in riparian forests of Benin (Natta 2003), 61.7% in gallery forests of the Hippopotamus Pond Biosphere Reserve at Burkina Faso (Bélem and Guinko 1998), and 70 to 75% in gallery forests at Lamto (southern Côte d’Ivoire) (Devineau 1975). The overall plant species composition makes EARF floristically comparable to the moist semi-deciduous forest of Nigeria (Lower Guinea) (Richards 1939) and the *Celtis* spp.-*Mansonia altissima* community of Côte d’Ivoire (Upper Guinea) (Guillaumet and Adjanohoun 1971). Guillaumet and Adjanohoun (1971) also pointed out that the dominance of Cannabaceae (previously Ulmaceae) and Malvaceae (previously Sterculiaceae) in the Upper and Lower Guinea forests offers evidence that West African semi-deciduous forests are at climatic climax. These indicator families were also reported as characteristics of the semi-deciduous forests in Ghana (Vooren and Sayer 1992) and Côte d’Ivoire (Swaine 1996) within the Upper Guinea zone. This high proportion of Guineo-Congolean species shows the floristic uniqueness of EARF and highlights its great importance in Benin which landscape is savanna dominated.

Unlike Palmer (1990) who showed that *Jack 1* is the most precise and least biased, it is rather the *Bootstrap* estimator (200.52 ± 9.2808) that seems to be closer to our field results (S = 185 species). The *Bootstrap* value is also close to estimates of Adomou et al. (2010) who assessed the specific richness of EARF around 200 species. This is what justifies the calculation of the four estimation methods (*Chao*, *first order jackknife*, *second order jackknife and bootstrap*) and not only one as Palmer (1990) would have demonstrated. In our study, the species accumulation curves were calculated with estimators (*Chao*, *Jack 1*, *Jack 2* and *Bootstrap*) and showed a change in species richness without flatten off at the right hand. The *Bootstrap* appears to be the best estimator which is closest to EARF plant richness estimated by Adomou et al. (2010). The Fig. 6 show that species accumulation curves were hardly tending towards the asymptote and are still climbing at the right hand end signifying that the sampling effort was insufficient. This suggests that the sampling did not capture all the species in EARF. The shape of the species accumulation curves should plateau for large numbers of sites sampled. However, the number of observed species will typically be smaller than the true number of species. Since species richness depends on sample size, we can expect that we will not have recorded all the species that occur in the survey area. So, the Species accumulation curves were used to ascertain range in values obtained with the different methods and we can expect that the correct total richness lies somewhere within that range. Many species will always remain unseen or undetected in a collection of sample plots. It is like the case of *Chrysophyllum welwitschii* (Sapotaceae) and *Drypetes aframensis* which, although reported in EARF (Adomou 2005), has not been recorded since 2014. *Drypetes aframensis* (Salicaceae) is also not mentioned in the Benin Flora. From a physiognomic point of view, it is important to underline that it is sometimes extremely difficult to survey some areas because of the thickness of the vegetation which can be very inaccessible. Other places are severely affected by human presence leading to very sparse vegetation or gap areas in the heart of the forest with completely isolated forest tree species. This is also the case of the periphery cleared by human boundaries encroachment and so many activities often carried out illegally. These constraints have often influenced the layout and the number of sampled sites. This also proves that despite the botanical sampling effort of Benin (Sosef et al. 2017), some priority species for conservation have either not yet been collected or reported and therefore could be omitted.

The connection of EARF with the West African forests blocks located on both side of the Dahomey Gap is emphasized here by the high proportion of Guineo-Congolean species recorded (66%). The high rate of phanerophytes and their phytochories testifies to the floristic originality of EARF in a crop and savanna dominated landscape. This justifies the physiognomic and floristic links of EARF with the two Guinean and Congolia forest blocks and substantiates the hypothesis that EARF is a remnant of the dense forests which were once a continuous block from west to central Africa as demonstrated by numerous palynological studies (Tossou 2002; Salzmann and Hoelzmann 2005) and also phylogeography (Demenou et al. 2016). The floristic link of the EARF with the West African semi-deciduous forest is also best highlighted by the high representation of many Upper Guinean endemic species belonging to the families of Cannabaceae (*Celtis mildbraedii*, *C. zenkeri* and *C. prantlii*), Malvaceae (*Triplochiton scleroxylon*, *Nesogordonia papaverifera*, *Mansonia altissima*, *Pterygota macrocarpa*, *Octobolus spectabilis* and *Sterculia tragacantha*), and Putranjivaceae (*Drypetes floribunda*, *D. gilgiana* and *D. aframensis*). This record provides strong evidence for past floristic connections with the West African rain forest zone which is consistent with paleovegetation reconstructions indicating that the tropical African rain forest formed a single block during the Holocene Humid Period (c. 9000–4500 yr BP) (Tossou 2002; Salzmann and Hoelzmann 2005; Demenou et al. 2018). Furthermore, the richness of EARF in *Rinorea* species (*R. batesii*, *R. brachypetala*, *R. dentata*, *R. kibbiensis* and *R. ilicifolia*) and their abundance are unique in the country. According to Akoègninou et al. (2006), *Rinorea batesii* Chipp, *Rinorea ilicifolia* (Welw. Ex OIiv.) Kuntze, *Rinorea dentata* are common in West, Central and East Africa and some of them in Madagascar. However, in Benin most of them are rare and are only found in EARF. *Rinorea brachypetala*, although it has been reported by Adomou (2005), was not mentioned in the National Flora (Akoègninou et al. 2006). *Rinorea* species are described as good indicators for West African semi-deciduous forests in a climax state (Achoundong 2000). The strong representation of *Rinorea* spp., the Cannabaceae (previously Ulmaceae) and the Malvaceae (previously Sterculiaceae) substantiate the view of Guillaumet and Adjanohoun (1971), who considered this forest type as the climatic climax or primeval type of semi-deciduous forest in West Africa. With this floristic composition, EARF can be seen as a unique West African rain forest refuge in a matrix of savanna-dominated vegetation in Benin.

**Figure 7.**
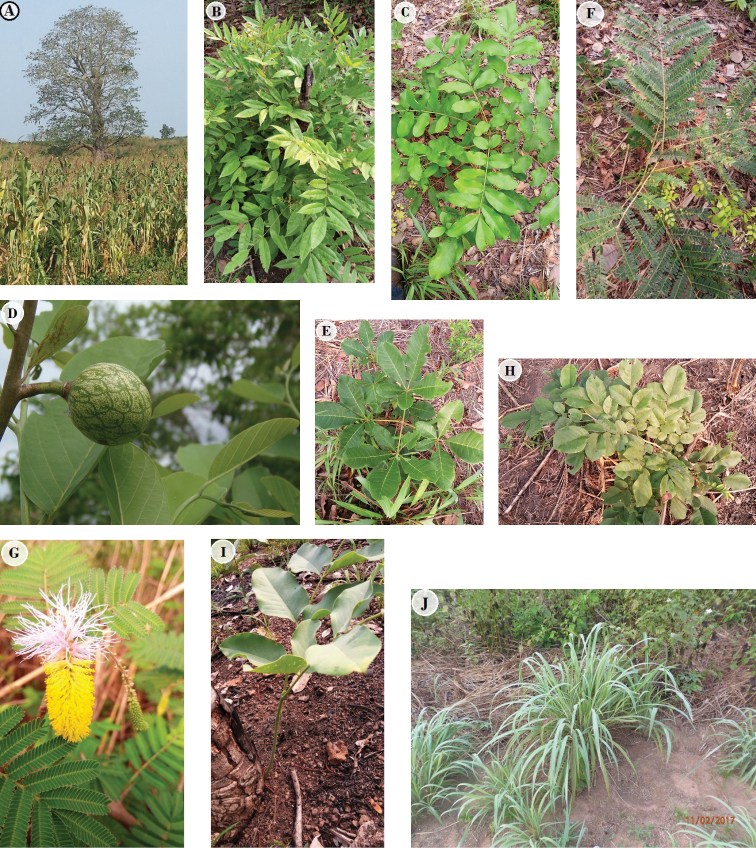
Species collected in the surrounding zone of Ewe-Adakplame Relict Forest **A***Adansonia digitata* (Samsung photo A. Houngnon 2015) **B***Pericopsis laxiflora* (Samsung photo A. Houngnon 2016) **C***Trichilia emetic* (Samsung photo A. Houngnon 2016) **D***Annona senegalensis* (Olympus photo A. Houngnon 2014) **E***Vitex doniana* (Samsung photo A. Houngnon 2016) **F***Parkia biglobosa* (Samsung photo A. Houngnon 2016) **G***Dichrostachys cinerea* (Samsung photo A. Houngnon 2016) **H***Pterocarpus erinaceus* (Samsung photo A. Houngnon 2016) **I***Daniellia oliveri* (Samsung photo A. Houngnon 2016) and **J***Andropogon gayanus* (Samsung photo A. Houngnon 2017).

**Figure 8.**
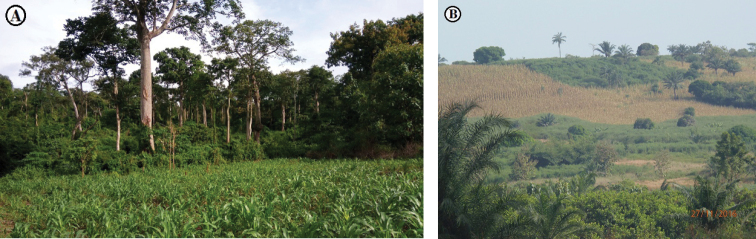
Ewe Adakplame Relict Forest in a matrix of savanna and agriculture landscape **A** forest edge affected by anthropogenic activities (Samsung photo A. Houngnon 2017) **B** Landscape of fallow and field around the edge of the forest ecosystem (Olympus photo A. Houngnon 2016).

In contrast, the surrounding vegetation at the immediate edge of EARF is composed of savanna species (Fig. 7A–J) from the Guineo-Sudanian transition zone such as: *Adansonia digitata* L. (Malvaceae), *Stereospermum kunthianum* Cham. (Bignoniaceae), *Trichilia emetic* Vahl (Meliaceae), *Annona senegalensis* Pers. (Annonaceae), *Vitex doniana* Sweet (Lamiaceae), *Parkia biglobosa* (Jacq.) R.Br. ex G. Don (Fabaceae-Caesalpinioideae (mimosoid clade)), *Dichrostachys cinerea* (L.) Wight & Arn. (Fabaceae- Caesalpinioideae (mimosoid clade)), *Pterocarpus erinaceus* Poir. (Fabaceae-Faboideae), *Pericopsis laxiflora* (Benth.) Meeuwen (Fabaceae-Faboideae), *Daniellia oliveri* (Rolfe) Hutch. & Dalziel (Fabaceae-Detarioideae), *Sarcocephalus latifolius* (Sm.) E.A. Bruce (Rubiaceae) and *Andropogon gayanus* Kunth (Poaceae). The landscape is characterized by two basin ecosystems with clear dissimilarity combining forest/savanna and crop areas in stable equilibrium (Figs 8A, B). This makes EARF a special site of rich biodiversity and emphasizes the vital role the forest plays as a corridor of transition conducive to resilience and the flow of genes for ecosystem equilibrium.

In total, we counted thirteen species restricted to one site in EARF. This is higher than the nine species previously reported by Adomou et al. (2010) in the EARF. Our record represents 15.4% of species with high conservation priority, thus describing EARF as being one of the richest sites in range-restricted plant species of Benin. This increase in range-restricted species can be explained by the fact that species with a high scarcity index are vulnerable and could disappear if biodiversity sanctuaries that protect them disappear (Juhé-Beaulaton 2010). Indeed, during the last three decades in Benin, some forests areas have been cleared with an annual degradation rate of 70,000 hectares per year (PNF Bénin 2004). This habitat loss has considerably narrowed the survival places where rare species were previously recorded.

Some of these species found in the single location of EARF within Benin (e.g., *Acroceras gabunense*, *Chrysophyllum welwitschii*, *Dovyalis afzelii*, *Drypetes aframensis*, *Drypetes gilgiana*, *Englerophytum oblanceolatum*, *Mansonia altissima*, *Nesogordonia papaverifera*, *Octolobus spectabilis*, *Pterygota macrocarpa*, *Rinorea ilicifolia*, *Rinorea kibbiensis* and *Vitex micrantha*) may gain more attention in the National Red List (Neuenschwander et al. 2011). Among them, there are many globally threatened species as the case of *Nesogordonia papaverifera* and *Mansonia altissima*, respectively reported as vulnerable (VU) and endangered (EN) by IUCN (2002) and later, were both assessed in Benin as critically endangered (CR) by Adomou et al. (2010) who considered EARF as sites with high concentration of threatened plant species in Benin. Moreover, the impact of harvesting on the survival of the most endangered species has been long ignored while many of them are not domesticated and many species uncharacterized. The case of *Mansonia altissima* is of urgent concern because its population is almost completely depleted, since it is locally used for roofing poles. *Englerophytum oblanceolatum* (Sapotaceae), which is not listed on the National Red List of Benin also tends to be concentrated in EARF (Houngnon 2014). Unfortunately, most of Benin Forest is still under severe threat due to expansion of towns, agricultural and fallow (Oloukoï et al. 2007) that are narrowing the natural habitat and leading to a massive loss of many of the local biodiversity taxa. To this end, since 2014, we have been trying to raise awareness among local communities from the villages of Ewe and Adakplame through participative action toward nursery establishment (Houngnon 2014) and vegetative propagation of native tree (Houngnon 2015) in order to rehabilitate the degraded lands and areas that could potentially be sensitive for the EARF durability.

## Conclusion

The importance of the flora of EARF testifies to its role in conserving forest biodiversity in the Dahomey gap corridor. This justifies its peculiarity and the relevance of this baseline vegetation information that could be used as complete range taxa that may allow us to test the forest refuge hypothesis against alternative speciation models across ecological gradients. As it happens, the management of the forest of this type, also raises the question of deepening interactions linking human environment in order to better understand the actual role that humans would have played in shaping ecosystems in the Dahomey gap since millennia. So, it would be interesting to understand the interplay between locals and EARF in order to explain its persistence in this savanna dominated landscape. Therefore, the actions to be considered following this checklist of EARF must take into account the community’s engagement in rehabilitating the degraded lands inside and around EARF.
